# Choking injuries: Associated factors and error-producing conditions among acute hospital patients in Japan

**DOI:** 10.1371/journal.pone.0267430

**Published:** 2022-04-27

**Authors:** Naomi Akiyama, Ryuji Uozumi, Tomoya Akiyama, Keisuke Koeda, Takeru Shiroiwa, Kuniaki Ogasawara

**Affiliations:** 1 School of Medicine, Iwate Medical University, Yahaba-cho, Iwate, Japan; 2 Department of Biomedical Statistics and Bioinformatics, Kyoto University Graduate School of Medicine, Kyoto, Japan; 3 School of Nursing, Iwate Medical University, Yahaba-cho, Iwate, Japan; 4 Economic Evaluation for Health (C2H), National Institute of Public Health (NIPH) Center for Outcomes Research, Wako, Japan; Mayo Clinic Rochester: Mayo Clinic Minnesota, UNITED STATES

## Abstract

Choking can lead to mortality and residual impairments. This study aimed to determine the factors associated with choking among acute hospital patients and examine error-producing conditions to suggest choking-prevention policies. Among 36,364 cases reported by hospital staff at an acute university hospital from 2012 to 2018 were examined using a retrospective study, 35,440 were analysis as the number of cases analysed for the study. We used descriptive statistics to present patient characteristics and conducted univariable and multivariable logistic regression analyses to identify factors associated with choking. Additionally, we conducted content analysis (root cause analysis) to examine error-producing conditions and prevention policies. Sixty-eight cases were related to choking injuries; of these, 43 patients (63.2%) were male, and 38 (55.9%) were aged 65 years and older. Choking cases had a high percent of adverse outcomes involving residual impairment or death (n = 23, 33.8%). Mental illness (adjusted odds ratio [95% confidence interval]: 3.14 [1.39−7.08]), and hospitalisation in the general wards (adjusted odds ratio [95% confidence interval]: 3.13 [1.70−5.76]) were associated with an increased probability of choking. Error production was caused by food (n = 25, 36.8%) and medical devices or supplies (n = 13, 19.1%). Almost all contributory factors were associated with inadequate checking (n = 66, 97.1%) and misperception of risk (n = 65, 95.6%). Choking poses a highly significant burden on patients, and hospital administrators should minimise the risk of choking to prevent related injuries. Hospital administrators should provide training and education to their staff and develop adequate protocols and procedures to prevent choking.

## Introduction

Medical errors threatens patient safety. The number of premature deaths in the US associated with preventable harm to patients has been estimated to be more than 400,000 each year [1]. The number of patient deaths caused by adverse events annually in Japan was estimated to be 1,326–1,433 [2]. Many people worldwide die each year due to preventable causes such as medical error.

Suffocation, including positional asphyxia and choking on food or other objects, was the third leading cause of death due to unintentional injury among adults aged 65 years and older from 2000–2013, accounting for 8% of unintentional injury deaths in the US [3]. Choking injuries are defined as the accidental swallowing of saliva, sputum, food and drink, or a lodged foreign object (edible or non-edible) in a person’s airway or oesophagus, which reduces or altogether obstructs airflow to the lungs [4, 5]. Choking may result in the clinical condition of suffocation or aspiration, and prolonged or complete choking results in asphyxia, leading to anoxia and even fatality [5]. The demand for long-term care for older adults is expected to rise sharply over the coming three decades [6, 7], and consequently, the prevention of choking should be emphasised. Choking is not only a problem among older adults but is also common among young children. Foreign-body aspiration is a cause of suffocation and continues to be a common problem in the field of paediatrics with potentially severe consequences, as it can result in both acute and chronic health problems [8, 9].

Previous studies have examined several factors associated with choking. First, some studies have investigated choking related to food and how age-related changes in eating and swallowing influence the risk of aspiration and choking [10]. Dementia, Parkinson’s disease, and pneumonitis are most strongly associated with food-related choking deaths among older adults [11]. Second, several studies have documented choking related to foreign bodies; paediatric research has indicated that airway restriction by foreign bodies most commonly occurs in young children, and almost 20% of children who have inhaled foreign bodies are between 0–3 years of age [12]. Research has also linked choking to disease and medication; asphyxia and choking are significant causes of mortality among people with mental illness, particularly those taking antipsychotic medication [13–16]. Intellectual disabilities and dysphagia are predictors of asphyxiation risk [17]. In addition, being young (aged 4–18 years), and having diseases that require emergency medical care are factors associated with increased mortality due to suffocation injuries [18]. Many previous studies have focused on factors associated with choking, especially in patients in the psychiatric and emergency departments and the general community. Hospital management should be aware of all possible injuries that can occur in hospital settings [19]. However, while there are several known risk factors for unexpected death from suffocation, these factors are yet to be thoroughly evaluated among general hospital patients [20]. Additionally, previous studies on choking focused on patient-related factors, and few have examined choking in relation to factors in the healthcare system, including clinical staff and risk management.

This study aimed to determine the factors associated with choking among acute hospital patients.

## Materials and methods

### Data source and collection

This was a retrospective study of incident reports from the fiscal years 2012–2018 collected by the medical safety management department at University Hospital A, an acute hospital with approximately 1,200 beds and 2,700 staff, including 500 physicians and 1,300 nurses. In Japan, the average length of stay in the wards, except psychiatric, long-term care, and tuberculosis wards, is 16.4 days [21]. In this study, an acute hospital patient was defined as a patient admitted to the critical care ward.

**Fig 1:**
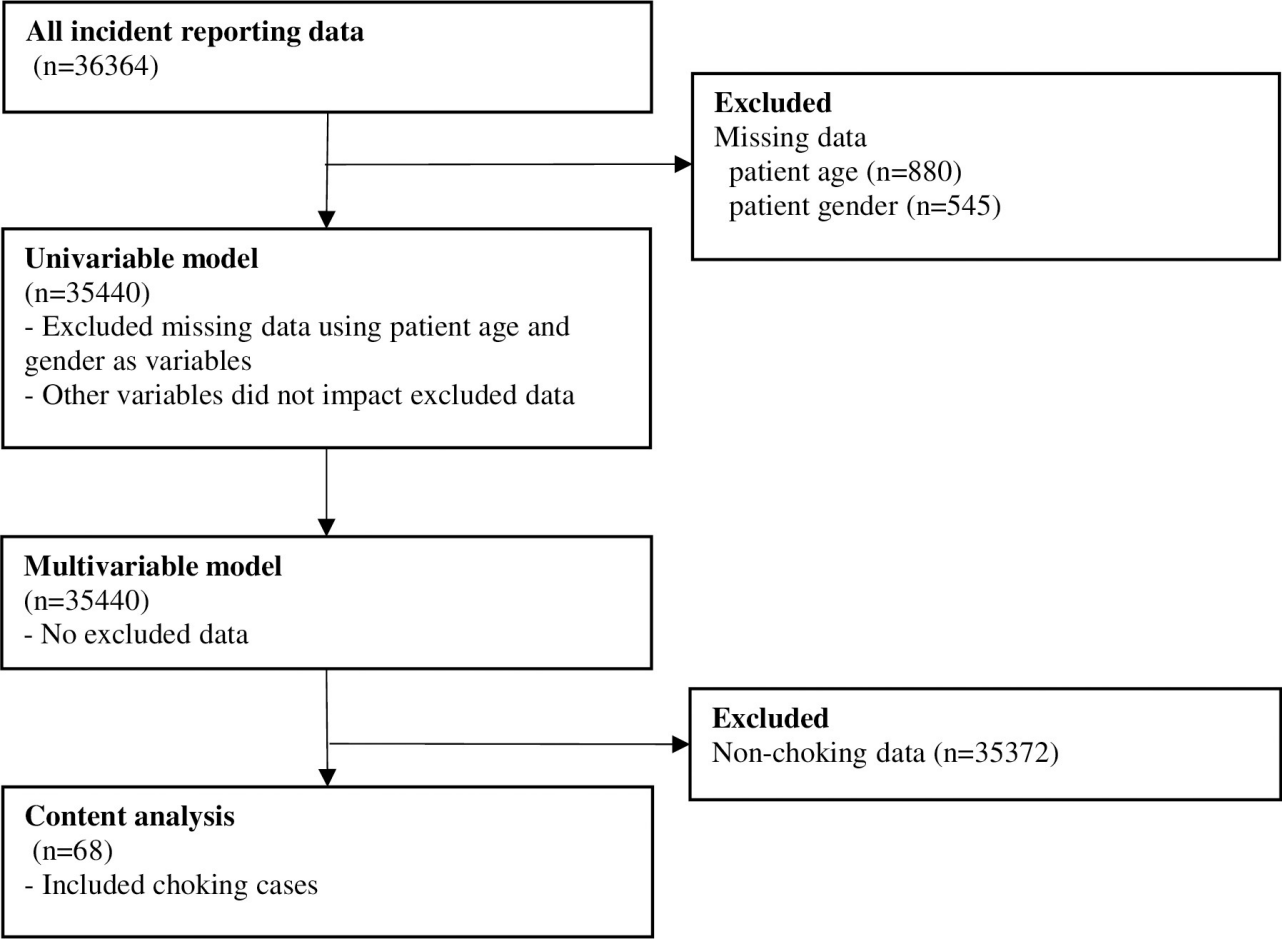
Flow diagram demonstrating the study process.

This study used secondary data collected from incident reports to examine the hospital’s medical safety measures. Hospital A requires the staff to report all types of preventable adverse events, errors, and near misses through an electronic reporting system. Errors are acts of commission (doing something wrong) or omissions (failing to do the right thing) that lead to undesirable outcomes or a significant potential for such outcomes. A near miss is defined as an error being committed, but the patient does not experience clinical harm, either through early detection or sheer luck [22]. Therefore, the scope for this review of incident reports included medical errors and near misses. The hospital administrator deleted personally-identifying information, such as patient names, identification numbers, and the names of hospital staff members, from the reports. The de-identified data were then made available to the research team. Incident-reporting data were sourced from 36,364 cases; we excluded cases with missing data and used 35,440 cases in the analysis, as shown in Fig 1.

### Ethical considerations

This study was approved by the ethics committee at the Iwate Medical University School of Medicine (approval number: MH2018-073). We obtained a comprehensive agreement from the participating hospital staff, including a notice posted on the hospital’s website. The notice included information about our study’s aim, the right to refuse participation in the research, the right to withdraw consent, and how we protect personal information. A third-party not involved in the research confirmed the data contained no personally-identifiable information and anonymised the data. The ethics committee approved obtaining comprehensive consent using the hospital website that the subjects often visit, rather than individual informed consent.

The recruitment period was from 31 October 2018–30 April 2019, during which time the participating hospital staff members could refuse the use of their data for this study if they so wished. The Trials Registry identification number for this study is UMIN-CTR (UMIN000041087), and the date of registration was 13 July 2020 (retrospectively registered).

### Study analysis

This study used a mixed-methods sequential explanatory design [23]. Qualitative methods were adapted to deepen our interpretation of the quantitative data, as the bias introduced by collecting one type of data may be mitigated by the collection of another type of data. We searched for terms such as ‘ingested’, ‘incorrectly ingested or aspiration’, ‘vomiting or suffocation’, ‘stuck in the throat’, and ‘choked on’ in the textual data of incident reports. After identifying potential choking cases, one of the authors, a registered nurse, assessed the identified cases. Cases where the patient had an underlying disease with aspiration pneumonia were excluded.

First, after conducting quantitative analysis, only cases of choking were selected, and their details were examined. Data related to case characteristics, including the patient’s age, gender, disability status, administrative area (outpatient, operation room, ward, or intensive care unit), year of occurrence of injury, and adverse outcomes were quantitatively analysed, while information regarding the medical error (details of error, background factors, reaction of the patient and their family, and additional treatment) was analysed as textual data in the qualitative analysis.

### Quantitative analysis

Quantitative data analysis was conducted using univariable and multivariable logistic regression models. For each case, choking served as the dependent variable and was binary (choking or other) in the logistic regression analysis. The characteristics, occurrence, and effect of choking on the patient were compared in choking and non-choking cases with univariable logistic regression analysis. Non-choking cases involved other medical errors, such as falls or medication or surgical errors. Next, a multivariable logistic regression analysis was conducted to investigate the factors associated with choking. Variables potentially associated with choking were selected based on prior research [11–16, 18], taking the number of events per variable into consideration based on the 1:15 rule [24]. We included the following variables: age, patient status (cognitive impairment, mental illness), and occurring situation. Additionally, since we were interested in patients in acute care wards, we focused on identifying whether the intensive care units and wards were associated with choking.

All analyses were performed using STATA version 14.0 for Windows (StataCorp). Odds ratios (ORs) and 95% confidence intervals (CIs) were presented from logistic regression analysis.

Patient age was entered as a continuous value. However, previous studies found that children and older adults are at a higher risk for choking; thus, we classified the data into three groups based on the patient’s age: <5 years, 5–64 years, and ≧65 years [3, 11]. Patient status indicated whether or not the incident reporter thought the patient had cognitive impairment or mental illness. It includes both the illness that resulted in hospitalisation and the medical history. We treated patient status as binary data; patient has cognitive impairment = 1 or none = 0, and patient has mental illness = 1 or none = 0. In the incident reports, based on the impact of medical errors, patients were classified into five categories: no disability, no potential of residual disability, low potential of residual disability, high potential of residual disability, and death. Since the sample size in this study was small, we focused on patients who had light residual impairment (i.e., no disability, no potential of residual disability, and low potential of residual disability) and those who had light treatment and high residual impairment or death (i.e., high potential of residual disability and death).

### Qualitative analysis

For the textual data, content analysis using root cause analysis (RCA) was conducted to examine the physical contributory factors, human or systemic contributory factors, and solutions for choking. Content analysis can be used with either qualitative or quantitative data, inductively and deductively [25]. RCA is a process used by hospitals to reduce the rates of adverse events and involves focusing on the factors contributing to the occurrence based on incident reports and determining potential ways of preventing reoccurrence [26]. Previous clinical studies have used RCA; for example, Aboumrad examined the factors contributing to cancer-related suicides [27]. In the present study, the textual data were coded referring to a study by Kellogg et al., which gathered and reviewed 302 self-reported adverse events [26]. Additionally, we referred to studies examining the human factors that influence the adverse events [28] and a report on food-related near misses and adverse incidents that caused the events [29].

The content analysis of textual data [30] was assigned to two nurse researchers, one of whom had experience as a patient safety administrator, whereas the other researcher was a nursing management specialist with experience as a nursing administrator and assistant to the director. Both were experienced in mixed-methods research and content analysis [31]. They independently read the incident reports on choking several times and divided them into meaningful units related to how and why. Meaningful units are a constellation of words or statements that have the same central meaning [30]. Specifically, the researchers searched for descriptions in the textual data for the ‘physical contributory factors of choking’, that is, why choking occurred or what objects were lodged in the airway. They also examined the ‘contributory factors related to choking’ in terms of why the choking occurred, its background factors, and the specific errors that contributed to choking. In this study, ‘contributory factors related to choking’ refers to the reasons the patient choked and the factors that caused it after, not prior to, admission. Based on physical and human or systemic contributory factors related to choking, a codebook was developed with reference to studies of human factors that contribute to errors [28, 32] and causes of choking events [29]. The codebook was then expanded with new codes for the current study, and the various codes were compared. The researchers discussed the tentative codes and identified the sub-codes and final codes. The sub-codes included the factors contributing to choking and solutions for those factors. The researchers discussed and selected the symbolic cases as examples of sub-codes for causal factors of choking and solutions to the contributory factors.

## Results

### Case characteristics of the cases studied

Choking and non-choking cases are compared in Table 1. Sixty-eight cases were complicated by choking, among which 38 patients (55.9%) were aged 65 years and older, and 10 patients (14.7%) were under the age of 5 years. Additionally, 43 patients were male (63.2%), and 25 were female (36.8%). Five patients (7.4%) had cognitive impairments and seven (10.3%) had mental illnesses. Regarding occurring situation, 2 (3.0%) in the intensive care unit and 53 cases (77.9%) occurred in a hospitalised in the general wards. The highest percentage of occurrence year was fiscal year 2018 (n = 24, 35.3%), followed by fiscal year 2015 (n = 14, 20.6%). In terms of the effect of patient adverse outcome, light residual impairment or light treatment was the highest (n = 45, 66.2%).

**Table 1:** Characteristics of choking cases.

			Choking n = 68	Others n = 35372
			n(%)	n(%)
Patient age				
	5 years ≦ age < 65		20(29.4)	13911(39.3)
	< 5 years		10(14.7)	3953(11.2)
	≧ 65 years		38(55.9)	17508(49.5)
Patient gender				
	Female		25(36.8)	15078(42.6)
	Male		43(63.2)	20294(57.4)
Patient Status				
	Cognitive impairment	Have	5(7.4)	1223(3.5)
		None	63(92.6)	34149(96.5)
	Mental illness	Have	7(10.3)	1261(3.6)
		None	61(89.7)	34111(96.4)
Occurring situation				
	Intensive care unit		2(3.0)	4270(12.1)
	Hospitalised in the general wards		53 (77.9)	16985(48.0)
	Others		13(19.1)	14117(39.9)
Occurrence year				
	Fiscal year 2012		5(7.4)	3226(9.1)
	Fiscal year 2013		9(13.2)	3425(9.7)
	Fiscal year 2014		6(8.8)	4276(12.1)
	Fiscal year 2015		14(20.6)	5334(15.1)
	Fiscal year 2016		2(2.9)	6072(17.2)
	Fiscal year 2017		8(11.8)	6124(17.3)
	Fiscal year 2018		24(35.3)	6915(19.5)
Adverse outcome				
	Light residual impairment or light treatment		45(66.2)	35084(99.2)
	High residual impairment or death		23(33.8)	288(0.8)

### Contributory factors for choking in logistic regression analysis

A logistical regression analysis was conducted to predict the physical factors contributing to choking in Table 2. There were differences between choking and non-choking patients in terms of their status regarding the following: mental disorder (crude OR [95% CI] = 3.10[1.42–6.80]) in univariable logistic regression model. A higher proportion of choking patients was hospitalised in the general wards (crude OR [95% CI] = 3.39[1.85–6.22]).

**Table 2:** Contributory factors for choking in logistic regression analysis.

				Univariable model			Multivariable model		
		n	Choking (%)	Crude OR	95% CI	p value	Adjusted OR	95% CI	p value
Patient age									
	5 years ≦ age < 65	13931	20(0.14)	ref.			ref.		
	< 5 years	3963	10(0.25)	1.76	[0.82–3.76]	0.145	2.73	[1.24–6.02]	0.013
	≧ 65 years	17546	38(0.22)	1.51	[0.88–2.60]	0.139	1.48	[0.84–2.60]	0.171
Patient Status									
	Cognitive impairment (ref. = none)	1228	5(0.41)	2.22	[0.89–5.52]	0.087	1.42	[0.55–3.68]	0.465
	Mental illness (ref. = none)	1268	7(0.55)	3.10	[1.42–6.80]	0.005	3.14	[1.39–7.08]	0.006
Occurring situation									
	Others			ref.			ref.		
	Intensive care unit	17038	53(0.31)	0.51	[0.11–2.25]	0.374	0.39	[0.08–1.74]	0.213
	Hospitalised in the general wards	4272	2(0.05)	3.39	[1.85–6.22]	0.033	3.13	[1.70–5.76]	<0.001

ref. = reference; OR = odds ratio; 95% CI = 95% confidence interval.

All four variables (patient age, cognitive impairment, mental illness and occurring situation) used in the univariable logistic regression model were included in the multivariable logistic regression models due to 68 chocking events. Choking and the following case characteristics were related; patient age ‘under 5 years old’ (adjusted OR [95% CI] = 2.73[1.24–6.02]), patient status of ‘mental illness’ (adjusted OR [95% CI] = 3.14[1.39–7.08]), and administrative area of ‘hospitalised in the general ward’ (adjusted OR [95% CI] = 3.13[1.70–5.76]).

### Physical contributory factors for choking

Table 3 shows the physical factors contributing to choking, which were, in order, food (n = 25, 36.8%), medical devices or supplies (n = 13, 19.1%), fluid (n = 12, 17.6%), daily necessities (n = 11, 16.2%), and medicine (n = 7, 10.3%). In terms of sub-codes, medical devices or supplies included medical thermometers, tubes (including tracheal intubation tubes or intravenous catheters), and oral case sponges. Daily necessities included shampoo and mouthwash. Medicine included anaesthetic, herbal medicine, and press-through packages.

**Table 3:** Physical contributory factors for choking.

Code	Example sub-codes
Food (n = 25, 36.8%)	Chunks of meat, milk, textural diet
Medical device/supplies (n = 13, 19.1%)	Thermometer, tube, medical tape, oral care sponge
Fluid (n = 12, 17.6%)	Vomit, sputum
Daily necessities (n = 11, 16.2%)	Shampoo, mouthwash, diapers
Medicine (n = 7, 10.3%)	Anaesthetic, herbal medicine, press-through package

^a^RCA, Root cause analysis.

^b^Number (%).

### Human or systemic contributory factors and solutions for choking

Table 4 shows the human or systemic contributory factors and solutions for choking. A total of 10 out of 68 (14.7%) cases resulted from a sudden turn in underlying disease and were not caused solely by choking. The most frequently-occurring errors were inadequate checking (n = 66, 97.1%) and misperception of risk (n = 65, 95.6%), followed by poor instructions or procedures (n = 51, 75.0%), inadequate coordination (n = 47, 69.1%), and inadequate environment (n = 29, 42.6%). Therefore, example solutions for the misperception of risk were: ‘*Don’t look away while the patient is eating to avoid suffocation by fast eating*’, ‘*Pay more attention using an observation system*’, and ‘*Conduct appropriate patient risk assessment before eating or fasting*’, which were coded as inadequate checking and poor instructions or procedure.

**Table 4:** Human or systemic contributory factors and solutions related to choking.

Code	Example sub-codes of solutions
Sudden turn in underlying disease (n = 10, 14.7%)	*・Not applicable as choking was unavoidable*
Inadequate checking (n = 66, 97.1%)	*・Do not look away while the patient is eating*
	*・Use a biological monitor*
Misperception of risk (n = 65, 95.6%)	*・Pay more attention using the observation system or visit the patient’s room more frequently*
	*・Conduct appropriate patient risk assessment before eating or fasting*
	*・Gain the ability to conduct a risk assessment*
Poor instructions or procedures (n = 51, 75.0%)	*・Conduct appropriate patient risk assessment before eating or fasting*
	*・Ensure appropriate patient/family education procedure*
Inadequate coordination (n = 47, 69.1%)	*・Keep a comfortable and safe position during eating or sleeping*
	*・Select appropriate texture of diet*
	*・Collect information about eating habits at home from the family*
Inadequate environment (n = 29, 42.6%)	*・Keep medical care equipment away from the patient*
	*・Move to a room that allows for easier observation*, *not a private room*
Lack of communication among staff (n = 19, 27.9%)	*・Share risk among staff using a written document containing the patient’s choking-associated factors*
Non-compliance with rules	*・Re-check the manual*
(n = 10, 14.7%)	*・Follow the doctor’s instructions*
Staff/time shortage/busyness (n = 8, 11.8%)	*・Manage time/staff*
Inadequate medication management (n = 7, 10.3%)	*・To avoid over-sedation*, *reschedule medicine time and provide adequate monitoring*, *or adjust dosage*
	*・Reconsider administration route or treatment*
Unfamiliarity with the task	*・Continue doing the task under supervision*
(n = 7, 10.3%)	
Educational mismatch of person and task (n = 7, 10.3%)	*・Obtain knowledge about the task*

^a^RCA, Root cause analysis.

^b^Number (%).

^c^Error-producing conditions were multiple choices.

Examples of inadequate coordination included the keywords ‘positioning’ and ‘textural diet supplies’, and example solutions included: ‘*Keep a comfortable and safe position during eating or sleeping to avoid suffocation*’ and ‘*Select an appropriately textured diet for patient’s swallowing abilities*, *and if necessary*, *collect information about eating habits at home from family*’.

The solution for an inadequate environment around the patient’s bed was to *keep medical care supplies away from the patient*. In cases where this was not ensured, when a staff or family member took their eyes off the patient, the patient reached their hands around their beds and swallowed or ingested medical devices or supplies or daily necessities, intentionally or unintentionally. The following most frequently cited error was lack of communication among staff (n = 19, 27.9%), and the most commonly proposed solution was ‘*Share risk among staff using a written document containing the patient’s choking-associated risk factors*’. Inadequate medication management affected seven cases (10.3%). In the case of medicine, over-sedation or anaesthesia led to respiratory depression and suffocation. Examples of sub-codes of solutions included: ‘*Reschedule medicine time and adequate monitoring to avoid respiratory depression by over-sedation*’ and ‘*Reconsider administration route or treatment to avoid suffocation*’.

## Discussion

This study investigated both patient- and healthcare-system-related factors leading to choking incidents and examined choking injuries in a large sample at an acute hospital. The results indicate that choking in the hospital had a severe effect on the patients and that most cases of choking were preventable. Additionally, choking was associated with patient characteristics and the characteristics of the hospital system, including human factors related to clinical staff, as human errors are individual and systemic problems.

Three major points were identified based on the findings of this study. First, choking did not occur in most cases, but when it did, the outcome was a severe impairment, including death. Second, a comparison between choking and other incidents suggested differences in the patient conditions and the situations in which they occurred. Specifically, the results of multivariate analyses suggested that children, mental illness in patients, and hospitalised in the general wards were the factors most strongly associated with choking. Meanwhile, the textual analysis revealed various physical contributory factors, including food or fluid, medical devices or supplies, and daily necessities. Third, most choking cases were preventable because the significant contributory factors were human errors related to misperception of risk and inadequate checking.

Our analysis revealed that choking had a severe impact on patient safety. In this study, choking occurred in 68 cases, representing 0.2% of the total data. However, choking occurred in 7.4% of all cases with high residual impairment or death. The same trend was observed in a previous study, where the overall mortality rate after suffocation injuries was 10.9% in US emergency departments [18]. In contrast, although over one-third of people over 65 years suffer from falls, injurious falls resulted in death in only 0.2% of the cases [33]. According to the present findings, although choking occurred infrequently, it had a severe impact on patient safety. Hospital administrators need to be aware of prevention policies, and the findings of a second and third surveys that have been planned will be helpful in the development of appropriate prevention policies.

This study revealed that patients’ conditions and the administrative area were related to choking. Additionally, choking was related to patients’ age; previous studies have indicated that children might be at an increased risk for foreign-body aspiration [8, 9]. In particular, mental illness and hospitalised in the general wards predicted a higher risk of choking. Mental illness remains a substantial risk factor for death by choking [13, 14], with a higher risk for those with schizophrenia and organic psychiatric illnesses, regardless of the medication administered [15]. Our findings from the acute hospital were similar to those of previous studies [13–15]. When patients are admitted to acute hospitals, the focus is on the patient’s main complaint, and even with a background of mental illness, admission to the ward may be based on the treatment of the main complaint rather than on the mental illness. Choking is one of the leading causes of accidental death among psychiatric patients [16] and is a common feature of psychiatric nursing, unlike in other wards, including general wards. Our findings suggest that the risk for choking is 3.1 times higher in patients with mental illness than in the general population. Raising risk awareness among all clinical staff, including general ward nurses, may inform the development of appropriate prevention policies. Our findings also show that choking was caused by food, fluid intake, medical devices, supplies, and daily necessities. The most common types of adverse events reported in previous studies were operative or surgery-related or associated with medication, drugs, or fluids [34, 35]. Many previous studies focused on food-related choking [10, 11] and foreign-body airway obstruction caused by coins or toys [36]. A study of deaths associated with choking in San Diego County reported that 133 victims had died from choking, but only 8 out of 133 deaths were caused by choking on non-food objects [37]. The participants of these previous studies were regular citizens, and the difference in the results may be attributed to the physical and mental conditions of patients admitted to an acute hospital. More factors related to patients need to be taken into account in a hospital setting than in community settings. Inpatients are often hospitalised in an unfamiliar environment, with a variety of underlying illnesses and disabilities, which complicate their needs. Therefore, clinical staff in the wards should be able to manage the likely risks successfully.

The integration of the results discussed thus far offers guidance for the prevention of choking. Reason [28], summarising the study of Williams [32], showed certain conditions related to both individuals and their immediate environment guaranteed to increase nominal error probabilities. Here, the error-producing conditions identified were ranked in the following order: misperception of risk, inadequate checking, and poor instructions or procedures. Although the reports were limited to food-related aspiration, the most common cause of the event was ‘neglected to observe’, followed by ‘misjudgement’, ‘neglected to check’, and ‘inadequate coordination’ [29]. These results were similar to our finding of a high probability for ‘misperception of risk’, ‘inadequate checking’, ‘inadequate environment’, and ‘inadequate coordination’. The Joint Commission identified inadequate hand-off communication as a contributory factor for adverse events, which is itself caused by many factors, such as healthcare provider training and expectations, language barriers, cultural or ethnic considerations, and inadequate, incomplete, or non-existent documentation [38, 39].

Recommendations for the prevention of choking and choking solutions include the ability of the clinical staff to perceive the adverse effects of choking. The staff must possess adequate training and education to be sufficiently equipped with situational awareness and adequate risk perception. Situational awareness is a predominant concern in system operations, based on a descriptive view of decision-making [40]. The World Health Organization has introduced a basic description of 10 topic areas related to organisational and human factors influencing patient safety, such as situation awareness tools and decision-making tools [41]. For a system that includes risk management by managers and hospital administrators, it is also necessary to provide clinical staff with opportunities to gain the ability to assess risk adequately and set up sufficient rules and procedures. A hand-off document is helpful as a communication tool for sharing information regarding a patient’s choking risk. However, at present, there are few guidelines for the prevention of choking. The National Patient Safety Agency found 605 reports of choking-related incidents in England and Wales involving adults with a learning disability, and developed guidelines explaining both risk factors and suggestions or guidance for health professionals, including adequate care [5]. The American Society of Anaesthesiologists’ task force on sedation and analgesia by non-anaesthesiologists developed the ‘Practice Guidelines for Sedation and Analgesia by Non-anaesthesiologists’ to allow clinicians to provide their patients with the benefits of sedation or analgesia while minimising the associated risks. It emphasised patient evaluation, monitoring, and the availability of emergency equipment in addition to other benefits [42].

With the increasing numbers of older patients being admitted to hospitals and subsequent increase in patients with dysphagia, the complete elimination of aspiration and choking is not realistic. While some choking injuries may not be preventable, as they occur in community settings, others are preventable through system prevention policies. For patient safety, it is essential to share knowledge of choking risk with clinical staff, the information management system, the patient, and the patient’s relatives. Hospital administrators and risk managers should develop evidence-based prevention policies to avoid patient choking injuries, and choking-related research needs to progress.

### Limitations

Our findings indicate that incident reports collected in a hospital are valuable data for predicting accidental injuries. However, this study has several limitations. First, the number of choking cases differed each year. However, since incident reports are reported on a voluntary basis, they may be underreported [43, 44]. We did not find an association between choking and older age in our study. However, a previous study reported that older adults were more likely to choke than young children and infants and were at an increased risk for aspiration pneumonia, which is strongly associated with mortality [10]. Furthermore, since recording near misses focused on not-occurring cases, the number of reports might vary depending on the reporter’s thought. Additionally, since we used retrospective and secondary data, our study depended heavily on the incident-reporting form. Data regarding the primary outcome, choking or other, were collected through a textual form by searching for typical terms and may therefore be undercounted depending on the incident reporter’s description. The incident-reporting system needs to establish a category of choking injuries to collect data for a prospective study.

Second, the data were from available samples. Since choking cases are rare, there is insufficient evidence regarding the risk factors; therefore, we used available samples. Long-term observations and surveys with more participating facilities are needed.

## Conclusion

Choking has become a social concern because it is often preventable. However, choking incidents continue to occur in hospitals. Our study revealed three significant findings. First, choking exerted a significant burden on patients, including severe residual impairment and death, underscoring the need for hospital administrators to minimise the risk of choking. Second, the quantitative and qualitative analysis revealed that the factors associated with choking were patients’ age, status, and ward admission, and contributing equipment included food or fluids and also medical equipment and daily necessities. Third, most of the human or systemic contributory factors of choking were misperceptions of risk by clinical staff. Individual clinical staff members need to gain the ability to assess risk through training and education. Additionally, introducing a risk assessment checklist for choking might be effective prevention.
